# Clinical Effects of Cigarette Smoking: Epidemiologic Impact and Review of Pharmacotherapy Options

**DOI:** 10.3390/ijerph14101147

**Published:** 2017-09-28

**Authors:** IfeanyiChukwu O. Onor, Daniel L. Stirling, Shandrika R. Williams, Daniel Bediako, Amne Borghol, Martha B. Harris, Tiernisha B. Darensburg, Sharde D. Clay, Samuel C. Okpechi, Daniel F. Sarpong

**Affiliations:** 1 Xavier University of Louisiana College of Pharmacy, 1 Drexel Drive, New Orleans, LA 70125, USA; asori12@gmail.com (D.L.S.); srwillia@xula.edu (S.R.W.); dannbetts@yahoo.com (D.B.); aborghol@xula.edu (A.B.); mharris1@xula.edu (M.B.H.); tbdarens@gmail.com (T.B.D.); shardeclay@hotmail.com (S.D.C.); 2 Department of Medicine, School of Medicine, Louisiana State University Health Sciences Center New Orleans, 1542 Tulane Avenue, New Orleans, LA 70112, USA; 3 Stanley S. Scott Cancer Center, School of Medicine, Louisiana State University Health Sciences Center New Orleans, 1700 Tulane Avenue, New Orleans, LA 70112, USA; samuelokpechi@gmail.com; 4 Center for Minority Health and Health Disparities Research and Education, Xavier University of Louisiana College of Pharmacy, 1 Drexel Drive, New Orleans, LA 70125, USA; dsarpong@xula.edu

**Keywords:** tobacco, cigarette, smoking, adverse health effects, non-pharmacologic therapy, pharmacotherapy

## Abstract

Cigarette smoking—a crucial modifiable risk factor for organ system diseases and cancer—remains prevalent in the United States and globally. In this literature review, we aim to summarize the epidemiology of cigarette smoking and tobacco use in the United States, pharmacology of nicotine—the active constituent of tobacco, and health consequence of cigarette smoking. This article also reviews behavioral and pharmacologic interventions for cigarette smokers and provides cost estimates for approved pharmacologic interventions in the United States. A literature search was conducted on Google Scholar, EBSCOhost, ClinicalKey, and PubMed databases using the following headings in combination or separately: cigarette smoking, tobacco smoking, epidemiology in the United States, health consequences of cigarette smoking, pharmacologic therapy for cigarette smoking, and non-pharmacologic therapy for cigarette smoking. This review found that efficacious non-pharmacologic interventions and pharmacologic therapy are available for cessation of cigarette smoking. Given the availability of efficacious interventions for cigarette smoking cessation, concerted efforts should be made by healthcare providers and public health professionals to promote smoking cessation as a valuable approach for reducing non-smokers’ exposure to environmental tobacco smoke.

## 1. Introduction

Tobacco use, in any form, can be described as a behavioral process which elicits psychological and physiologic addictive mood among users. Nicotine, the active ingredient in tobacco, is highly addictive, resulting in sustained tobacco use. Tobacco use is divided into combustible and noncombustible tobacco products. Combustible tobacco products include: cigarettes, cigars, cigarillos, small cigars, water pipes (hookah), and pipes. Noncombustible tobacco products include electronic cigarettes and tobacco formulations developed for chewing, dipping, or snuffing.

According to the 2013–2014 National Adult Tobacco Survey (NATS), the United States’ national prevalence for current tobacco product use was 21.3% in adults aged ≥18 years [1]. Distribution of tobacco product use include: 17% for cigarettes, 1.8% for cigars/cigarillos/filtered little cigars, 0.3% for pipes, 0.6% for water pipes/hookah, 3.3% for electronic cigarettes, and 2.5% for smokeless tobacco [1]. These trends sharply contrast with tobacco use in the 1800s—a period which saw predominant use of chewing tobacco and pipe tobacco because there was no mass manufacturing of cigarettes. These less popular methods of tobacco use, while still unhealthy, theoretically were associated with fewer cancers and tobacco related deaths. Now, with its unique design and accessibility, cigarette smoking has become the choice of tobacco use among many youth and adults globally. Cigarettes are designed to allow deep inhalation of smoke into the lungs, delivering high levels of nicotine to the brain within 10–20 s of inhalation [2]. This rapid rise in nicotine levels makes cigarette smoking the most reinforcing and dependence-producing form of tobacco use [2]. The epidemiologic impact and adverse health effects of cigarette smoking are significant. Reducing the prevalence of cigarette smoking and the resultant smoking-induced disease is imperative.

This article reviews the epidemiology of cigarette smoking in the United States, pharmacology of nicotine, and health impact of cigarette smoking alongside behavioral and pharmacological interventions available for smoking cessation in the United States. We performed a literature search on Google Scholar, EBSCOhost, ClinicalKey, and PubMed databases using the following keywords in combination or separately: cigarette smoking, tobacco smoking, epidemiology in the United States, health consequences of cigarette smoking, pharmacologic therapy for cigarette smoking, and non-pharmacologic therapy for cigarette smoking. We reviewed and included literature that provided the most relevant and up-to-date information on our search terms. Excluding the epidemiology data, which focused on the United States, our literature search was inclusive of literature without any geographic constraint. The aim of this review is to provide accessible information on the clinical effects of cigarette smoking, interventions available for cigarette smoking cessation, and the cost estimates for U.S. Food and Drug Administration (FDA)—approved pharmacotherapy options for cigarette smoking.

## 2. Epidemiology of Cigarette (Tobacco) Smoking in the United States

Although cigarette smoking is the most commonly used form of tobacco in the U.S., the prevalence of cigarette smoking amongst adults has been declining in recent years. According to the 2015 National Health Interview Survey (NHIS), the percentage of adults aged ≥18 years who smoked cigarettes was 15.1% in 2015, a decrease from 20.9% in 2005 [3]. This general trend of decline in tobacco smoking in the United States has also been observed globally [4]. The World Health Organization (WHO) reports that among adults over 15 years, the global rate of smoking declined from 23.5% in 2007 to 20.7 in 2015, reflecting a 2.8% smoking rate reduction [4]. Although there has been a decline in the prevalence of smoking globally, the number of people smoking worldwide has remained at 1.1 billion from 2007 to 2015 because of population growth [4]. Several factors linked to declines in the prevalence of smoking include population-based interventions such as raising tobacco taxes, tobacco price increases, anti-tobacco mass media campaigns, comprehensive smoke-free laws, enhanced access to help quitting tobacco use, and implementation of governmental regulations of tobacco products [1,4,5]. Of all these factors, WHO reports that raising tobacco taxes has been the single most effective way to reduce tobacco use [4,5].

The result of the NHIS also highlights several disparities in the prevalence of cigarette smoking [3]. Cigarette (tobacco) smoking is more prevalent among adult males than adult females [3]. The prevalence of cigarette smoking in 2015 was 16.7% among adult males and 13.6% among adult females [3]. Prevalence was highest among adults aged 25–44 years (14.8%) and lowest among persons aged ≥65 years [3]. Racial and ethnic differences also exist. The prevalence was highest amongst American Indian/Alaska Natives (21.9%), and lowest among Asians (7.0%) [3]. When examining education level, prevalence was variable. It was highest among those with a General Education Development Certificate (GED) (34.1%) and lowest among those with a graduate degree (3.6%) [3]. When examining socioeconomic status, prevalence was highest among persons living below poverty level (26.1%) and lowest among persons living at or above poverty level (13.9%) [3]. 

A history of substance abuse disorders and mental illness increases cigarette smoking [6,7]. Cooperman et al. reported a high prevalence of cigarette smoking (80%) among opiate dependent smokers on methadone treatment and Santhosh et al. disclosed a 2013 report which showed that although patients with mental illness and substance abuse disorders made up 24.8% of adults in the United States, they consumed nearly 40% of all cigarettes [6,7]. Additional data from the National Surveys on Drug Use and Health corroborate the strong association among cigarette use, mental illness, and substance abuse across gender and age [7].

Cigarette (tobacco) smoking is not only common among adults, but is also common among youth. With the current trends of monetary investment into the tobacco industry, smoking poses a bigger threat to the younger population in American society. According to the Executive Summary of the U.S. Surgeon General Office report in 2012, everyday 3800 youth under the age of 18 start smoking [8]. Most adult smokers, 88%, smoked their first cigarette before the age of 18 [8]. According to the National Survey on Drug Use and Health 2012, the mean age of smoking initiation was 15.3 years and less than 1.5% of cigarette smokers began smoking in adulthood (after 26 years of age) [9]. Although cigarette smoking most often begins during youth and young adulthood, the use of cigarettes among this population has been declining in recent years. Among high school students, 9.3% reported current cigarette smoking in 2015, a decrease from 15.8% in 2011 [10]. Among middle school students, 2.3% reported current cigarette smoking in 2015, a decrease from 4.3% in 2011 [10]. While the use of cigarettes among youth has declined, the use of electronic cigarettes in this population is increasing. Electronic cigarettes are currently the most commonly used form of tobacco among middle and high school students. In 2015, 16% of high school students reported current electronic cigarette use, an increase from 1.5% in 2011 [10]. Among middle school students, 5.3% reported current electronic cigarette use, an increase from 0.6% in 2011 [10]. These trends in cigarette and electronic cigarette use highlight the importance of targeting smoking prevention efforts at youth and young adults.

Electronic cigarettes (e-cigarettes) are rapidly increasing in popularity [11]. There was an increase in e-cigarette use from 1.9% in 2012–2013 to 3.3% in 2013–2014 according to the National Adult Tobacco Survey [1]. Young adults between 18–24 years account for the highest prevalence of use of newly emerging products, including e-cigarettes and water pipes/hookahs [1]. E-cigarettes use a battery-powered heating device to deliver nicotine via a vapor that is drawn into the mouth, upper airways and possibly lungs [11]. The device uses a battery-powered heating element activated by suction or manually to heat a nicotine solution and transform it into vapor [11]. In a study by D’Ruiz et al., they observed that e-cigarettes had blood plasma nicotine levels lower than that of conventional tobacco cigarettes, yet the reduction in craving was comparable between e-cigarettes and conventional tobacco cigarettes [12]. E-cigarettes usually contain nicotine dissolved in a solution made up of propylene glycol and/or glycerin, and flavorings [12,13,14]. Other toxic substances such as formaldehyde and acrolein may be present in very low levels in e-cigarettes compared to conventional cigarettes [14]. Although the use of e-cigarette is soaring, several review articles evaluating studies of e-cigarettes have concluded that the short- and long-term effects of e-cigarettes are limited or lacking [13,14]. Even with limited data on the health effects of e-cigarettes, in 2016, the U.S. Food and Drug Administration (FDA)—under authority granted to it by the Congress under the Family Smoking Prevention and Tobacco Control Act of 2009—took a historic step to protect America’s youth from the harmful effects of using e-cigarettes by extending its regulatory authority over the manufacturing, distribution, and marketing of e-cigarettes [15].

Although current gaps exist in scientific evidence on the spectrum of health effects of e-cigarettes, we know that compared with older adults, brain of youth and young adults is more vulnerable to the negative consequences of nicotine exposure [15]. These effects include addiction, priming for use of other addictive substances, reduced impulse control, deficits in attention and cognition, and mood disorders [15]. The U.S. Surgeon General in his report on “E-Cigarette Use Among Youth and Young Adults: A Report of the Surgeon General” raised awareness on the exponential growth of youth and young adults who are using e-cigarettes and encourages concerted societal effort to prevent and reduce the use of e-cigarettes by youth and young adults in order to prevent the well documented harmful effects of nicotine use-which is more pronounced in the development of adolescent brain [15].

The CDC also discusses the potential for harm and benefit associated with e-cigarette use [16]. E-cigarettes can cause harm to the public, which is more notable if used by defined populations (youth, young adults, pregnant women). Some of the harms include increased risk for using nicotine and/or other tobacco products, leading former smokers to relapse to nicotine and/or tobacco product use, delay smoking cessation among current smokers, exposure to second-hand aerosol, and nicotine poisoning [16]. E-cigarette use also contributes to environmental tobacco smoke and may mimic the effects of passive (second-hand) smoking seen with use of conventional cigarettes [17]. Potential benefit of e-cigarette is that it can help us transition our society to little or no combustible tobacco use [16]. There is also emerging data suggesting that e-cigarettes may facilitate smoking cessation but further research is needed to compare the effectiveness and safety of e-cigarettes compared to other nicotine replacement therapies [13,14]. Consistent with the U.S. FDA regulatory oversight and the U.S. Surgeon General report, it may be prudent to investigate further the health effects of e-cigarettes prior to widespread advocacy favoring its use as a replacement for combustible tobacco use, given that the public health effects of e-cigarettes are yet to be fully understood.

## 3. Pharmacology of Nicotine

**Figure 1:**
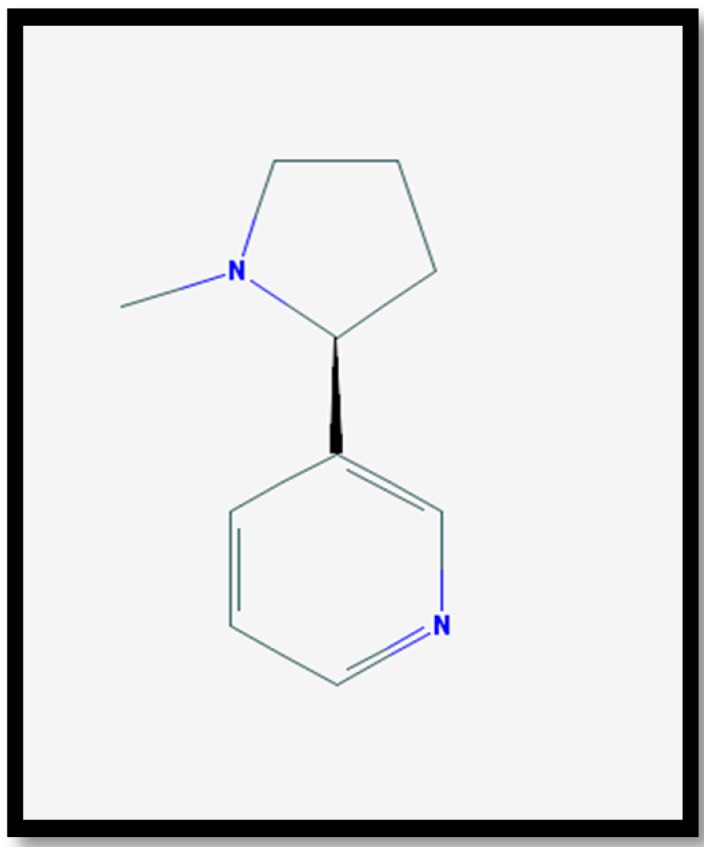
The chemical structure of nicotine [23].

Nicotine (C_10_H_14_N_2_)—see Figure 1—is a plant alkaloid found in the tobacco plant and is the principal constituent of tobacco responsible for its addictive character [18,19]. Nicotine acts as a ganglionic nicotinic cholinergic agonist in the autonomic ganglia, brain, spinal cord, neuromuscular junctions and adrenal medulla [18,20,21]. Nicotine has dose-dependent pharmacological effects and has both stimulant and depressant action [20,22].

The effects of nicotine on the central nervous system (CNS) and its peripheral stimulating effects are mediated through the release of several neurotransmitters, including acetylcholine, beta-endorphin, dopamine, norepinephrine, serotonin, and adrenocorticotropic hormone (ACTH) [18]. Notable stimulant effects of nicotine stimulant activities include peripheral vasoconstriction, elevated blood pressure, tachycardia, increased cardiac output, and enhanced mental alertness and cognitive function [18,20,22]. Depressant effects of nicotine include muscle relaxation and anxiety reduction [20,22]. At higher doses, nicotine stimulates the “reward” center in the limbic system of the brain [20].

Nicotine use produces a feeling of pleasure and relaxation [20]. In dependent smokers, the urge to smoke cigarettes correlates with a low blood nicotine level, as though smoking were a means to achieve certain nicotine level, reap the rewarding feeling associated with nicotine and avoid withdrawals [22]. Repetitive exposure to nicotine leads to neuroadaptation and building of tolerance to nicotine’s initial effects [20]. Accumulation of nicotine in the body leads to a more substantial withdrawal reaction if cessation is attempted [20]. Common withdrawal symptoms include anxiety, difficulty concentrating, irritability, and strong cravings for tobacco [20]. Onset of these withdrawal symptoms occurs within 24 h and can last for days, weeks, or longer [20]. Nicotine replacement therapies neither achieve the peak levels seen with cigarettes nor produce the same magnitude of subjective effects of cigarette smoking, they do, however, suppress the symptoms of nicotine withdrawal [22]. 

Nicotine from cigarette is carried on inhaled tar particles into the lungs where a large alveolar surface area allows rapid absorption into the pulmonary circulation [21]. Nicotine is well distributed with a volume of distribution of about 2.6 L/kg [21]. It undergoes primarily hepatic (80–90%) metabolism—with the remainder of the metabolism taking place in the lungs and kidney—to inactive metabolite: cotinine. Nicotine has a half-life of 1–4 h and about 2–35% is excreted unchanged in the urine [21].

## 4. Health Effects of Cigarette (Tobacco) Smoking

Annually, more than 400,000 individuals die prematurely in the United States from cigarette use; this represents almost one of every five deaths in the United States [9]. Approximately 40% of cigarette smokers will die prematurely due to cigarette smoking unless they are able to quit [9]. Between 1965 and 2014, over 20 million Americans died either from chronic conditions caused by smoking or exposure to secondhand smoke, complications caused by smoking during pregnancy, or smoking related fires in residential buildings [9]. Table 1 outlines the common causes of smoking-related deaths between 1965 and 2014 [9].

**Figure 2:**
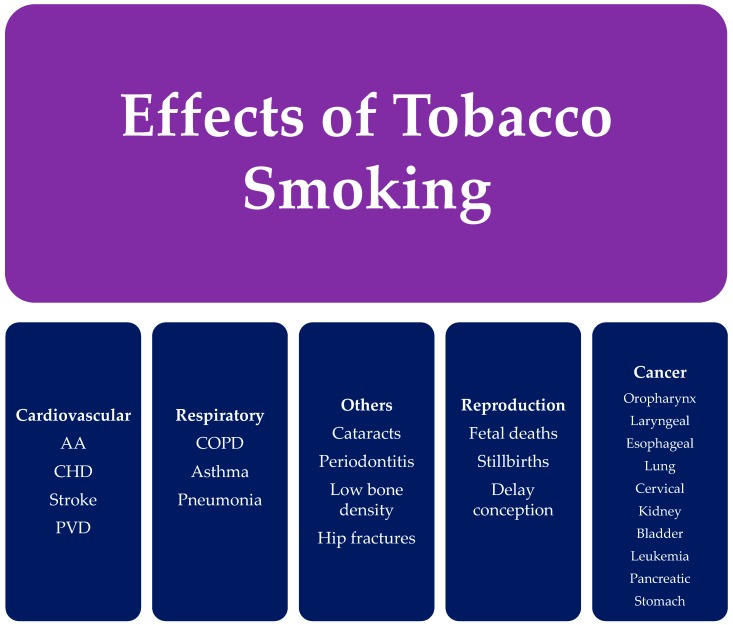
Effects of tobacco smoking [25]. (AA) Aortic aneurysm; (CHD) Coronary heart disease; (PVD) Peripheral Vascular Disease; (COPD) Chronic obstruction pulmonary disease.

Cigarette smoking affects the human body in myriad ways, causing the development of chronic diseases and cancers. Figure 2 categorizes common health effects of tobacco smoking. The health effects are seen not only in smokers, but also individuals exposed to secondhand smoke. The impact of cigarette smoking on health depends on the duration of smoking over years and the exposure to cigarette (tobacco) smoke. The mechanism by which cigarette (tobacco) smoke causes adverse health outcomes involves multiple complex steps resulting from the exposure to free radicals from the components of tobacco smoke, leading to increased oxidative stress, inflammation, and DNA damage [9]. The chemical toxins in tobacco smoke are transferred from the lungs to the blood stream, where it is transported to nearly every part of the human body.

### 4.1. Cancer

Smoking is currently the largest preventable cause of cancer-related deaths, accounting for approximately 30% of cancer related deaths [24]. Carcinogens in cigarette smoke bind to human DNA, resulting in DNA damage and gene mutations. These genetic changes lead to uncontrolled cell growth and inhibit normal mechanisms that restrain cell growth and spread, resulting in cancer. A causal relationship has been established between cigarette (tobacco) smoking and lung cancer, the leading cause of cancer-related deaths in the U.S. There is also a causal relationship between cigarette smoking and cancers of the head, neck, liver, bladder, cervix, esophagus, colon, and rectum [9]. The evidence is insufficient to conclude that there is a causal relationship between smoking and cancers of the breast and prostate, however there is an increased risk of dying from cancer in smokers with breast, prostate, and other cancers [9].

### 4.2. Cardiovascular Diseases

There is a causal relationship between cigarette smoking and cardiovascular events. Major mechanisms underlying smoking-induced cardiovascular disease include endothelial dysfunction, prothrombotic effects, inflammation, altered lipid metabolism, increased demand for myocardial oxygen and blood, decreased supply of myocardial blood and oxygen, and insulin resistance [9]. Cigarette smoking and exposure to second hand smoke are major causes of coronary heart disease, stroke, aortic aneurysm, and peripheral arterial disease [25]. Cigarette smoking and secondhand smoking are also a major cause of death due to CVD. Annually, 194,000 deaths from cardiovascular disease in the U.S. are smoking-related [9].

### 4.3. Respiratory Diseases

Cigarette (tobacco) smoking is also associated with the development of chronic pulmonary diseases. In fact, cigarette smoking is the primary cause of COPD in the U.S. [26,27]. Some of the mechanisms involved are loss of cilia in the lungs, mucus gland hyperplasia, and overall inflammation resulting in the abnormal functioning of the lungs as well as injury. Cigarette smoking may exacerbate asthma in adults. Underlying mechanisms may include chronic airway inflammation, impaired mucociliary clearance, increased bronchial hyperresponsiveness, increased development of T helper cell 2 (Th2) pathways relative to Th1 pathways, increased production of IgE, and greater allergic sensitization [9,25]. Smoking also increases the risk of developing tuberculosis and dying from tuberculosis [9].

### 4.4. Reproductive Effects

Maternal cigarette (tobacco) smoking causes several reproductive abnormalities. Carbon monoxide in cigarette smoke binds to hemoglobin, depriving the fetus of oxygen, ultimately resulting in low birth weight [25]. Other toxins in tobacco smoke including nicotine, cadmium, lead, mercury, and polycyclic aromatic hydrocarbons, have been found to cause sudden infant death syndrome, premature births, and decreased fertility in women [9,24]. More recent evidence indicates a causal relationship between maternal cigarette smoking and orofacial clefts and ectopic pregnancies [9]. A causal relationship between smoking and erectile dysfunction in men has also been established [9].

### 4.5. Additional Effects

Smoking impairs immune function, resulting in an increased risk of pulmonary infections and rheumatoid arthritis [9]. It also affects the gastrointestinal tract, increasing the risk of peptic ulcer disease. There is also increased risk of hip fractures and low bone mineral density in postmenopausal women who smoke. Additionally, smokers with diabetes have a higher risk of developing complications, including nephropathy, blindness, peripheral neuropathy, and amputations [25]. Recent evidence indicates that the risk of developing type 2 diabetes is 30–40% higher in smokers that nonsmokers [9]. Passive (second-hand) smoking has also been linked with negative health consequences such as low-birth rate in offspring of mothers exposed to second-hand smoke, sudden infant death syndrome, and type 2 diabetes mellitus [28].

## 5. Non-Pharmacologic Treatment of Cigarette (Tobacco) Smoking

About 70% of cigarette smokers visit a physician each year [29]. Even more smokers visit pharmacists, dentists, nurses, and other healthcare professionals. Clinicians are, therefore, in an excellent position to identify smokers. It is recommended that tobacco use of every patient treated in a healthcare setting be assessed and documented at every visit [29]. Identifying smokers in the healthcare setting offers a good opportunity for clinicians to recognize and guide effective interventions for smoking cessation [30]. Patients who begin any major behavioral or lifestyle change go through successive stages of change. To plan an effective intervention, it is important to understand these major stages of change [26]. Intervention strategies should target the individual’s current stage of change, with an initial objective of moving the individual to the next stage and an overall goal of moving the individual to the maintenance stage. Table 2 below reviews the stages of change.

A simple five-step algorithm called the 5 A’s (Ask, Advise, Assess, Assist, Arrange) can be used by clinicians to offer a brief counseling intervention in the primary care setting [29]. The 5 A’s are concisely described in Table 3. Some of the myriad reasons that patients may be unwilling to quit are as follows. They may be unaware of the harmful effects of tobacco or do not understand the benefits of quitting. They may not have the financial resources to facilitate the smoking cessation process. Also, they may have fears or concerns about quitting, or may be demoralized because of failed quit attempts. Patients in this category or others who are unwilling to quit may respond to brief motivational interventions that are based on principles of Motivational Interviewing (MI) [29]. The 5 R’s of smoking cessation summarize the areas that should be addressed in Motivational Interviewing (MI). The 5 R’s are described in Table 4.

Specific non-pharmacologic interventions for smoking cessation can be categorized into three approaches: clinical approaches, public health approaches, and alternative approaches. Clinical approaches to smoking cessation include self-help programs, telephone counseling, cognitive-behavioral approaches such as individual and group counseling, healthcare provider interventions, and exercise programs. Public health approaches include community-level interventions, workplace interventions, multimedia interventions, and public policy changes [31]. Alternative approaches include acupuncture, aversive therapy, and hypnosis. These various interventions are briefly discussed in Table 5. It is also important to understand barriers to smoking cessation and effectively address these barriers using motivational intervention technique. A systematic review by Twyman et al. reported common barriers to smoking cessation [32]. The review found that smoking for stress management, lack of support from health and other service providers, and the high prevalence and acceptability of smoking in vulnerable communities were three consistent barriers to smoking cessation common to six-select vulnerable groups (low socioeconomic status, indigenous, mental illness and substance abuse, homeless; prisoners; and at-risk youth) [32]. Knowledge of the barriers to smoking cessation and implementation of methods to address these barriers are imperative in helping patients quit smoking.

## 6. Pharmacologic Treatment of Cigarette (Tobacco) Smoking

All patients who are trying to quit smoking should be offered pharmacologic intervention except when these medications are contraindicated or in certain populations where there is insufficient evidence of effectiveness (e.g., pregnancy, adolescence, light smokers) [29]. Pharmacologic therapy should be used in addition to behavioral support for smoking cessation [36]. There are seven FDA approved medications for smoking cessation: transdermal nicotine patch, nicotine gum, nicotine lozenge, nicotine inhaler, nicotine nasal spray, bupropion sustained-release (SR), and varenicline. These medications should be considered first line therapy according to the U.S. Public Health Service guidelines [29]. First line agents are summarized in Table 6. Patients who do not respond to any first line medications or who have contraindications to first line agents may be prescribed second line agents. Second line agents include clonidine and nortriptyline. Second line agents are not FDA approved for smoking cessation but have demonstrated some effectiveness in treating tobacco use [37]. Combination therapy of pharmacologic agents is often used in patients who have failed to achieve cessation with monotherapy. Combination therapy involves adding short acting nicotine replacement therapy (nicotine gum, lozenge, inhaler, or nasal spray) to longer acting agents, such as the nicotine patch or bupropion SR [37]. Table 7 includes the wholesale acquisition cost of the FDA approved smoking cessation therapy for consideration by providers and patients. Clinicians tasked with selecting appropriate pharmacologic therapy for smoking cessation should consider using the first line agents prior to considering the second line therapy, except when there are contraindications to first line agents or when patients did not respond to first line therapy. Clinicians should also consider other factors such as cost, adverse effect profile, and route of medication delivery. The goal of therapy should be to administer an affordable agent with proven efficacy and good tolerability profile. Selecting a medication formulation that helps patients to achieve medication adherence is also desirable.

### 6.1. Nicotine Replacement Therapy (NRT)

Five nicotine replacement therapy (NRT) products are approved by the U.S. Food and Drug Administration for tobacco dependence treatment: nicotine gum, nicotine lozenge, nicotine nasal spray, nicotine inhaler, and the transdermal nicotine patch [38]. The nicotine inhaler and nasal spray are prescription drugs in the U.S., whereas the nicotine gum, lozenge and patch are available over the counter. NRTs work to reduce severity and duration of withdrawal symptoms by partially replacing nicotine obtained by tobacco use. A 2008 meta-analysis of 69 clinical trials found that all five nicotine replacement products are superior to placebo, approximately doubling abstinence rates [39]. A Cochrane Review of 150 trials also found that all forms of nicotine replacement therapy (inhaler, oral tablets/lozenges, gum, patch, and nasal spray) increased rates of quitting smoking by 50–70% [40]. A study enrolling 504 patients found that all forms of NRT evaluated (gum, patch, nasal spray, and inhaler) produced similar quit rates and were equally effective at reducing the frequency, duration, and severity of urges to smoke [41]. NRT is generally well tolerated with mild adverse effects. The three most commonly reported adverse effects of NRT in observational studies were headache, nausea and vomiting, and other gastrointestinal symptoms [38,42]. Adverse effects of NRT are generally formulation specific, depending on the delivery system used [38,42]. NRT must be used with caution in patients with known cardiovascular conditions, but have generally found to be safe in patients with these conditions [38,42]. All products are pregnancy category D with the exception of the nicotine gum (category C), although the benefit of replacement therapy may outweigh the risks [37,43].

### 6.2. Bupropion Sustained-Release (SR)

Bupropion is the first non-nicotine agent to demonstrate efficacy in the treatment of tobacco dependence [37]. Bupropion sustained-release is FDA approved for smoking cessation and is regarded as a first line therapy by the U.S. Public Health Service guideline [29]. Bupropion is an inhibitor of dopamine and norepinephrine reuptake, but its mechanism of action in smoking cessation is not well understood [37]. A systematic review of 44 clinical trials, published in 2014, found that sole therapy with bupropion significantly increased long-term (≥6 months) smoking abstinence (RR = 1.62; 95% CI, 1.49–1.76) [44]. The most common adverse effects with bupropion, when used for smoking cessation, are insomnia, which occurs in about 30–40% of patients, and dry mouth, which occurs in 10% of patients [37]. A more serious side effect is seizure, which can occur because bupropion reduces the seizure threshold. Two large studies reported seizure incidence of 0.1% [37]. Bupropion has a boxed warning for development of neuropsychiatric symptoms ranging from agitation to suicidal ideation and behavior in patients using this medication [45]. In 2009, the FDA issued an alert to healthcare professionals reporting that cases of neuropsychiatric symptoms have occurred in patients without pre-existing psychiatric illness and have worsened in patients with pre-existing psychiatric illness [45]. The FDA recommends close monitoring of neuropsychiatric symptoms in patients receiving Bupropion and to stop Bupropion therapy when necessary and to monitor patient closely until neuropsychiatric symptoms resolve [45]. Bupropion is pregnancy category C and has been shown to be safe and effective in patients with known cardiovascular conditions [46,47]. 

### 6.3. Varenicline

This is a first line agent for smoking cessation. Varenicline is a partial agonist specific for the neuronal nicotinic acetylcholine receptor subtype α_4_β_2_. As a partial agonist, it binds to and produces partial stimulation of the nicotinic receptor, thereby reducing the symptoms of nicotine withdrawal [37]. Varenicline also stimulates dopamine turnover, which provides relief from nicotine cravings and withdrawal symptoms that can occur when a patient is trying to quit [37]. A 2008 meta-analysis found that varenicline increased the odds of quitting three times than that of placebo (OR 3.1, 95% CI 2.5–3.8) and produced a quit rate of 33 percent at six month follow-up [29]. In a systematic review of 39 clinical trials comparing nicotine partial agonists, varenicline significantly increased smoking abstinence at 6 months or longer compared to placebo (RR = 2.24; 95% CI, 2.06–2.34) or bupropion (RR = 1.39; 95% CI, 1.25–1.54). [48] A 2008 meta-analysis also found varenicline to be superior to placebo (OR 2.55; 95% CI, 1.99–3.24) and bupropion (OR 2.18, 95% CI, 1.09–4.08) [39]. Varenicline is generally well tolerated, with the most common adverse events being nausea, insomnia, and headache [37]. Varenicline can be used in patients who have concurrent CVD, but with caution. It should be noted that in 2011 the FDA published a warning, based on data from a clinical trial of smokers with CVD which stated that “cardiovascular adverse events were infrequent overall, however, certain events, including heart attack, were reported more frequently in patients treated with Chantix^®^ (Varenicline) than in patients treated with placebo” [49,50]. Varenicline is pregnancy category C and, like bupropion, carries a black box warning for increased risk of behavior change, agitation, depressed mood, and suicidal ideation and behavior [49,50,51].

### 6.4. Clonidine

Clonidine should be used as a second line agent when primary therapies are found to be ineffective. Clonidine is only FDA approved for hypertension but has shown to be efficacious in smoking cessation. Clonidine is a α_2_-adrenergic agonist, whose effect in smoking is thought to be based on its ability to counteract CNS features of nicotine withdrawal, including craving and anxiety. Results from a Cochrane Review article found that clonidine approximately doubled the rate of abstinence compared to placebo (OR, 1.89; 95% CI, 1.30–2.74) [52]. Clonidine is limited by its adverse effect profile, which includes postural hypotension, extreme drowsiness, fatigue, and dry mouth [37].

### 6.5. Nortriptyline

Nortriptyline should be used as a second line agent when primary therapies are found to be ineffective. Nortriptyline is a tricyclic antidepressant, whose effects in smoking cessation are not well understood. A meta-analysis review of 6 randomized clinical trials indicated that nortriptyline treatment doubles the odds of smoking cessation, with an OR for abstinence of 2.1 (95% CI, 1.5–3.1) [53]. The most common side effects of nortriptyline are related to its anticholinergic effects, including dry mouth, constipation, and sedation [37].

## 7. Conclusions

Although its prevalence has declined in recent years, cigarette smoking remains the most common method of tobacco use. The adverse health effects associated with cigarette smoking are numerous; thus, continual efforts to reduce the prevalence of cigarette smoking are imperative. Current trends on cigarette smoking highlight the importance of smoking prevention and smoking cessation initiatives that target youth. Promotion of smoking cessation can be a strong public health approach for reducing non-smokers’ environmental exposure to environmental tobacco smoke. Treating tobacco dependence should include both behavioral and pharmacologic interventions. First line agents for smoking cessation include bupropion SR, varenicline, and nicotine replacement therapies. 

One of the goals of Healthy People 2020 is to “reduce the illness, disability, and death related to tobacco use and secondhand smoke exposure” [55]. Twenty-one national objectives, related to tobacco use, are outlined to achieve this goal. Recommended strategies for achieving this goal include: increasing the cost of tobacco products; fully funding tobacco control programs; banning smoking in public places; anti-tobacco media campaigns, particularly those targeted towards youth; community, school, and college anti-tobacco programs; encouraging and assisting tobacco users to quit; expanding insurance coverage of smoking cessation agents; and expanding state quit line capacity [55,56].

## Figures and Tables

**Table 1:** Premature deaths caused by smoking and exposure to secondhand smoke, 1965–2014 [9].

Cause of Death	Total
Smoking-related cancers	6,587,000
Cardiovascular and metabolic diseases	7,787,000
Pulmonary diseases	3,804,000
Conditions related to pregnancy and birth	108,000
Residential fires	86,000
Lung cancers caused by exposure to secondhand smoke	263,000
Coronary heart disease caused by exposure to secondhand smoke	2,194,000
Total	20,830,000

**Table 2:** Stages of behavior change [26].

Stages of Change	Description
Pre-contemplation	The patient is not yet ready to quit at this time or within six months
Contemplation	The patient is considering quitting at some point in the future, but has not yet taken any action towards quitting.
Preparation	Patient is planning to quit in the next 30 days
Action	Patient is in the process of quitting or has quit within the last six months.
Maintenance	The patient has quit smoking for at least three months.

**Table 3:** The “5A’s” model for treating tobacco use and dependence [29].

Intervention	Description
Ask	Implement a system to ensure that all patients are asked their tobacco use status at every visit.
Advise	Urge every tobacco user to quit. Advice should be clear, strong, and personalized Clear—“I think it is important for you to quit smoking now and I can help you.” Strong—“As your clinician, I need you to know that quitting smoking is the most important thing you can do to protect your health now and in the future.” Personalized—“Continuing to smoke makes your asthma worse, and quitting may dramatically improve your health.”
Assess	Assess every tobacco user’s willingness to quit smoking. If the patient is willing to make a quit attempt, provide assistance. If the patient will participate in an intensive treatment, deliver or refer treatment. If the patient clearly states he or she is unwilling to quit provide motivational intervention. If the patient is a member of a special population (e.g., adolescent, pregnant smoker), provide information specific to that population.
Assist	Provide aid for the patient to quit. This includes: ▪ Forming a quit plan. ▪ Recommending the use of pharmacologic therapy, if indicated. ▪ Providing practical counseling ▪ Providing social support ▪ Providing supplementary materials including websites and quit-lines that will assist in cessation.
Arrange	Schedule follow-up contact, either in person or by telephone. Follow-up contact should occur soon after the quit date, preferably during the first week. A second follow-up contact is recommended within the first month. Schedule further follow-up contacts as indicated.

**Table 4:** Enhancing motivation to quit tobacco—The “5 R’s” [29].

Intervention	Description
Relevance	Motivational information to a patient is more effective if it is personally relevant to a patient
Risk	The acute and long-term risks of smoking should be stressed. It is most effective if smoking can be tied to the patient’s current health or illnesses and the health of others
Rewards	Encourage the patient to identify potential benefits of smoking (e.g., Improved health, saving money, etc.)
Roadblocks	Ask the patient to identify barriers or impediments to quitting and provide treatment that address these barriers
Repetition	Repeat the motivational intervention each time an unmotivated smoker visits the clinic

**Table 5:** Non-pharmacologic interventions for smoking cessation [31,33,34,35].

Studies	Intervention	Description	Efficacy
	**Clinical Approaches**		
Niaura [31]	Self-help programs	Printed or electronic materials given to patients to increase motivation and provide advice to quit.	Generic materials: OR, 1.24 (95% confidence interval (CI): 1.07–1.45); Tailored materials: OR, 1.42 (1.26–1.61) (based on 11 trials with ≥6-month follow-up)
	Telephone counseling	Quit hotlines or using a counselor to call patients.	OR, 1.56 (95% CI: 1.38–1.77) (based on 27 trials with ≥6-month follow-up)
	Cognitive-behavioral therapy Individual	Individual or group sessions that focus on addressing and changing thinking and behavior in smokers.	OR, 1.56 (95% CI: 1.32–1.84) (based on 21 trials with ≥6-mo follow-up)
	Cognitive-behavioral therapy Group		OR, 2.17 (95% CI: 1.37–3.45) (based on 55 trials with ≥6-month follow-up)
	Healthcare provider interventions	Advice given to patients from clinicians during routine contact.	Meta-analysis of 37 studies with a mean sample size of 507 each, physician advice had the greatest impact on increasing cessation (*p* = 0.002)
	Exercise programs	Exercise based interventions	OR, 2.36 (95% CI: 0.97–5.70) (based on 1 trial with 12-month follow-up)
	**Public Health Approaches**		
Niaura [31]	Community-level interventions	Include various approaches such as distribution of “quit kits”, support groups, smoke-free areas, and others.	COMMIT trial demonstrated modestly higher odds of quitting only in light smokers (less than 25 cigarettes/day) in an intervention community compared with a control community (OR, 1.17; *p* < 0.05)
	Workplace interventions	Include various approaches such as seminars, online interventions, and others.	Meta-analysis of 19 studies demonstrated significantly improved odds of abstinence at 6 and 12 months, but not thereafter
	Multimedia interventions	Use differing multimedia such as internet, videos, to aid in cessation.	Large scale campaign in NY that used education, referrals, school-based programs, and poster contests resulted in an absolute decrease in smoking prevalence of 10% over the 5-year study period
	Public health policy [ 31]	Include smoking bans	Ban of all public smoking in Italy resulted in a 2.3% decrease in smoking prevalence <1 year. later
	**Alternative Approaches**		
White, et al. [33]	Acupuncture	Involves penetration of the skin with needles to stimulate certain points on the body.	Meta-analysis of 33 randomized trials found no differences in long-term abstinence rates for acupuncture
Hajek, et al. [34]	Aversive therapy	Increasing the amount of smoking over time with the goal of inducing a sense of displeasure.	Meta-analysis of 25 randomized trials found insufficient evidence to support a clear dose-response relationship between aversive therapy and smoking cessation
Barnes, et al. [35]	Hypnosis	Creates unconscious change in patients undergoing hypnosis in the form of new thoughts or attitudes.	Systematic review of 11 randomized trials found insufficient data to support the use of hypnotherapy for smoking cessation

**Table 6:** Pharmacologic agents for smoking cessation [29,30,37,38].

Generic Name	Brand Name(s)	Mechanism of Action	Common Adverse Effects	Dose
Nicotine gum	Nicorette, Equate, Top Care, others	Partially replace the nicotine formally obtained from tobacco, which aids smoking cessation by reducing the severity of withdrawal symptoms and cravings	Jaw pain, mouth, soreness, dyspepsia, hiccups	The 2-mg gum is for patients smoking less than 25 cigarettes/day; the 4 mg gum for patients smoking 25 or more cigarettes/day. Use at least 1 piece every 1 to 2 h for the 1st 6 weeks; the gum should be used for up to 12 weeks with no more than 24 pieces to be used per day
Nicotine lozenge	Sunmark, Top Care, others	See above	Mouth and throat, hiccups	2 mg lozenge for patients who smoke their 1st cigarette more than 30 min after waking, and the 4 mg lozenge for patients who smoke their 1st cigarette within 30 min of waking. Generally, smokers should use at least 9 lozenges/day in the first 6 weeks; the lozenge should be used for up to 12 weeks, with no more than 20 lozenges/day
Nicotine patch	Nicoderm CQ, Equate, others	See above	Mild skin irritation at placement site	For those who smoke more than 10 cigarettes/day: 21 mg patch for 6–8 weeks, decrease 14 mg for 2–4 weeks, then 7 mg for 2–4 weeks. For less than 10 cigarettes/day: 14 mg for 6 weeks, decrease to 7 mg for 2–4 weeks.
Nicotine inhaler	Nicotrol	See above	Mouth and throat irritation, cough	A dose from consists 1 inhalation. Recommended dosage is 6–16 cartridges/day. Recommended duration of therapy is up to 6 months.
Nicotine nasal spray	Nicotrol NS	See above	Runny nose, throat and nasal irritation, cough	Spray 1–2 doses/h, increasing as needed for symptom relief. Minimum recommended treatment is 8 doses/day, with a maximum of 40 doses/day (5 doses/h). Each bottle contains approximately 100 doses. Recommended duration of therapy is 3–6 months
Bupropion SR	Zyban, Wellbutrin SR	Inhibitor of dopamine and norepinephrine reuptake, but its mechanism of action in smoking cessation is not well understood	Insomnia, dry mouth, headache, tremors, nausea, anxiety	Begin treatment 1–2 weeks. quit date. Begin with a dose of 150 mg every morning for 3 days, then 150 mg twice daily. Dosage should not exceed 300 mg/day. Dosing at 150 mg twice daily should continue for 7–12 weeks. For long-term therapy, consider use for up to 6 months post-quit
Varenicline	Chantix	Partial agonist specific for the neuronal nicotinic acetylcholine receptor subtype α_4_β_2_.	Nausea, insomnia, abnormal dreaming, headache	Start 1 week before the quit date at 0.5 mg once daily for 3 days, then 0.5 mg twice daily for 4 days, then 1 mg twice daily for 3 months approved for up to 6 months. Note: Patient should be instructed to quit smoking on day 8, when dosage is increased to 1 mg twice daily

**Table 7:** Smoking cessation medications and cost [54].

Drug	Some Available Formulations	Usual Adult Maintenance ^a^ Dosage (Max Dose)	Cost ^b^
**Nicotinic Receptor Agonists**			
Nicotine polacrilex gum-eneric	2, 4 mg/piece	48 mg/day, 96 mg/day	$151.79–$205.99
Nicorette Gum (GSK)			$173.91–$195.64
Nicotine polacrilex lozenge-generic	2, 4 mg/lozenge	40 mg/day, 80 mg/day	$224.07 ^c^
Nicorette Gum (GSK)			$232.43 ^c^
Nicotine transdermal patch-generic	7, 14, 21 mg/24 h patches	1 patch/day ^d^	$49.78 ^c,e^
Nicoderm CQ (GSK)			$52.32 ^c,e^
Nicotine nasal spray—Nicotrol NS (Pharmacia & Upjohn)	200 sprays/10 mL bottle (0.5 mg/spray)	1 dose (2 sprays) 40mg/day (max 5 doses/h) ^f^	$333.69 ^g^
Nicotine oral inhaler—Nicotrol (Pharmacia & Upjohn)	10 mg cartridges	16 cartridges/day	$317.80 ^h^
**Dopaminergic-Noradrenergic Reuptake Inhibitors**			
Bupropion SR-generic	100, 150, 200 mg SR tabs ^j^	150 mg bid ^k^	$27.00
Wellbutrin SR (GSK) ^i^			$377.00
Zyban			$235.97
**Nicotinic Receptor Partial Agonist**			
Varenicline—Chantix (Pfizer)	0.5, 1 mg tabs	1 mg bid ^l^	$337.23

^a^ Dosage reduction may be needed for hepatic or renal impairment. ^b^ Appropriate WAC for 30 days’ treatment at the maximum usual maintenance dosage. WAC = wholesaler acquisition cost, or manufacturer’s published price to wholesalers. WAC represents a published catalogue or list price and may not represent an actual transactional price. Source: Red Book Online^®^ System (electronic version). Truven Health Analytics, Greenwood Village, Colorado, USA. Available at: http://www.micromedexsolutions.com/ (cited: 10/10/2016). ^c^ Same price for all dosages. ^d^ See specific label for instructions for dose titration. ^e^ Cost for 28 transdermal patches. ^f^ One spray per nostril. Maximum of 40 doses/day should not be used for >3 months. ^g^ Cost of four 10-mL bottles. ^h^ Cost of 168 10-mg cartridges; each cartridge delivers 4 mg of nicotine. ^i^ Not FDA-approved for this indication. ^j^ Only the generic 150 mg SR tablets are FDA-approved for this indication. ^k^ Initial dosage is 150 mg once/day for 3 days. ^l^ Initial dosage is 0.5 mg once/day for 3 days, then bid for 4–7 days.
